# Colorectal Cancer and Its Screening Among Public in the Western Region of Saudi Arabia

**DOI:** 10.7759/cureus.27404

**Published:** 2022-07-28

**Authors:** Khalid M Alzahrani, Shouq M Fallatah, Rahaf A Almehmadi, Jana S Alghamdi, Abeer I Alsulaimani, Layla M Alkhaldi, Ali G Alsuwayhi

**Affiliations:** 1 General Surgery, Taif University, Taif, SAU; 2 College of Medicine, Taif University, Taif, SAU; 3 Internal Medicine, King Faisal Medical Complex, Taif, SAU

**Keywords:** risk factor, screening, western region, saudi arabia, symptoms, colorectal cancer

## Abstract

Background: Colorectal cancer (CRC) is defined as a cancer that starts in the colon or the rectum. CRC is the most commonly diagnosed cancer among Saudi males and ranks third in female individuals. Awareness among the population about CRC symptoms, risk factors, and screening tests is essential for preventing further morbidity and mortality. This study aimed to assess CRC knowledge in the western region of Saudi Arabia, as well as awareness of CRC risk factors, symptoms, and screening based on various demographic data.

Methods: We conducted a cross-sectional study with a representative random sample of 358 Saudi residents in the western region. A self-administered questionnaire was distributed through social media sites starting from October 2021 till December 2021. Participants' awareness of CRC risk factors, symptoms, and screening was assessed using the questionnaire. For data analysis, we used SPSS Statistics, version 24 (IBM Corp., Armonk, NY).

Results: Of the 385 participants, 76.4% were females, and most participants had a university degree level of education. Study respondents reported fear of colonoscopy as the top reason why they avoided CRC screening. Most participants (63.4%) showed insufficient knowledge. Certain factors showed a significant association with the participants' knowledge about CRC, such as age (p<.001), higher education (p=.002), and having a career in health care (p=.002).

Conclusion: As the study resulted in overall insufficient knowledge about CRC among respondents, certain factors showed a significant association with the knowledge level. Raising awareness and health promotion programs should target older age groups and those with below university degree level of education to ultimately prevent morbidity and mortality related to colorectal cancer.

## Introduction

Colorectal cancer (CRC), according to the American Cancer Society, starts in the colon or the rectum. It may also be called colon cancer or rectal cancer [1]. CRC is described by histopathology as medullary carcinoma or adenocarcinoma, which is the most common histological type, with mucinous and undifferentiated signet-ring adenocarcinoma comprising around 10% of all types [2].

Colorectal cancer is ranked as one of the most common cancers globally, just third to lung and breast cancers, and is considered the fourth leading cause of cancer-related deaths (approximately 0.88 million deaths annually) [3]. Colorectal cancer is the second most common cancer among women and the third most common among men worldwide [4]. The average risk of colorectal cancer in Saudi Arabia is 9.6 per 100,000 population with a median diagnosis age of 55 years in women and 60 years in men [5]. In 2014, CRC was ranked second to breast cancer among Saudi female and first among all male malignancies, presenting with a sharp increase in the incidence over the past few years. During 1994-2004, the Saudi Cancer Registry reported that almost 44.6% of patients diagnosed with CRC in Saudi Arabia have a chance of five-year survival [5].

Regarding clinical presentation, persistent changes in bowel habits, hematochezia, chronic abdominal pain, weakness and fatigue, fever, melena, and unexplained weight loss are all symptoms of CRC, and patients may present with one or more of these clinical manifestations. Additionally, iron deficiency anemia (IDA) with no clear cause, fatigue, dyspnea or orthostatic dizziness, irritable bowel syndrome, inflammatory bowel disease, infection, and hemorrhoids are some of the differential diagnoses that might be mistaken for CRC [1]. There are several risk factors for CRC that can be classified into modifiable risk factors such as smoking, obesity, lack of physical activity, alcohol, low-fiber diet, high consumption of red meat, and low fruit and vegetable intake. Non-modifiable risk factors include aging, gender, prior colon diseases such as inflammatory bowel disease, diabetes type 2, family history of CRC, and polyps [6]. Colorectal cancer is a multifactorial condition; however, it is one of the preventable diseases [1]. Unfortunately, in Saudi Arabia, the majority of CRC cases are diagnosed during clinical evaluations rather than through screening programs.

CRC screening tests are essential tools that can detect the disease early, thus aiding in preventing the disease. Among all cancers, CRC is ideal for screening due to its high incidence rates and long duration times between early and advanced stages [7]. Therefore, CRC screening plays a crucial role in decreasing the disease incidence and reducing the mortality rate through detecting a higher proportion of cancers at an earlier stage, resulting in more options of treatment. CRC screening is required and recommended for all people over the age of 50. Screening by colonoscopy is carried out once every 10 years, while screening by sigmoidoscopy is recommended once every five years, and fecal occult blood test is carried out once annually; however, colonoscopy remains the standard screening method in CRC. Nevertheless, patients at risk of CRC, such as those having first-degree family relatives with CRC, should have their screening started 10 years before the age when the first-degree family relative was diagnosed [7]. It is essential to determine the suitability and cost-effectiveness of these screening tests among Saudi patients [5]. The American Cancer Association recently updated the screening guidelines, setting the age at which colorectal cancer screening should begin to 45 years for average-risk individuals [1].

In the literature, several barriers to CRC screening were reported by the general population including personal fears, economic problems, lack of awareness about the symptoms, signs, risk factors, and outcome of the disease, the benefits of screening, and lack of doctor recommendations [4]. Increasing the awareness among people about lifestyle modification and recognizing warning signs and symptoms may be important to reduce CRC morbidity and mortality [8]. Therefore, if CRC is diagnosed at an early stage, the survival rate may reach to more than 90%, according to the American Cancer Society [1].

Unfortunately, the literature lacks research that discusses the general population knowledge and awareness about CRC risk factors, symptoms, and screening methods along with the associated factors. Therefore, we conducted this study to assess these aspects of knowledge regarding CRC among the Saudi Arabia western region population based on several demographic factors.

## Materials and methods

Study design

This was an analytical cross-sectional study that utilized a self-administered electronic questionnaire that was distributed through social media from October 1, 2021, to December 31, 2021. The study used a convenient sampling technique to include the general population aged 18 years or older living in the western region of Saudi Arabia. Those with a history of inflammatory bowel disease were excluded. The required sample size was 385 calculated using an online sample size calculator Raosoft (Raosoft, Inc., Seattle, WA) with a confidence level set at 95% and a margin of error of 5%.

Data collection tool and technique

An electronic self-reported questionnaire was developed by the study authors based on the available literature and was face-validated by experts in the field [5]. The questionnaire acquired participants' sociodemographic data and family history of CRC, and assessed their knowledge about CRC risk factors, symptoms, and screening. To calculate participants' knowledge score, each correct answer was considered equal to one point, and the highest achievable score was 34 points. After calculating the total score for each participant, the scores were categorized into insufficient knowledge, if the score was 17 points or less, and sufficient knowledge, if the score was 18 points or higher.

Ethical consideration

Ethical approval was obtained from the research ethics committee of Taif University (application number HAO-02-T-105; from November 2021 to November 2022) before data collection. An informed consent was obtained from each participant after explaining the study in full and clarifying that participation was voluntary. Data collected were securely saved and used for research purposes only.

Statistical analysis

Data were entered and analyzed using IBM SPSS Statistics for Mac, version 24 (IBM Corp., Armonk, NY). All categorical variables were summarized as frequencies and percentages. For bivariate analyses, the chi-square test was used to analyze categorical data. A p-value of <.05 was considered statistically significant.

## Results

A total of 385 participants were included and their data analyzed. Table 1 shows that most participants (38.7%) fell in the 25- to 40-year-old age category. The majority of the respondents were female representing 76.4% of the sample, and 77.7% of the participants were holding a university level degree. Moreover, most participants (79.2%) had a career that was not related to the health care discipline. The geographic distribution showed that 47.3% of the participants were from Taif city, which represented the majority, followed by Makkah city with 21.3% of the study respondents. Only 19.2% of the participants had a family member or a friend with a history of CRC; meanwhile, a large proportion of 67.2% individuals had heard about CRC in general (Table 2). As shown in Table 2, participants with a family member or close friend with a history of CRC had heard more about CRC than other participants (p=.002).

**Table 1:** Demographic characteristics of participants (total n=385) CRC: colorectal cancer

Characteristics		n	%
Age (years)	18-24	136	35.3%
	25-40	149	38.7%
	41-50	71	18.4%
	>50	29	7.5%
Gender	Male	91	23.6%
	Female	294	76.4%
Education	University degree level	299	77.7%
	Below university degree level	86	22.3%
Career related to health care	Related to health care	80	20.8%
	Not related to health care	305	79.2%
Region of residence	Taif	182	47.3%
	Makkah	82	21.3%
	Jeddah	57	14.8%
	Medina	53	13.8%
	Yanbu	11	2.9%
Family or close friends had a CRC history	Yes	74	19.2%
	No	311	80.8%
Have you ever heard of CRC?	Yes	272	70.6%
	No	113	29.4%
Have you ever heard of any screening tests that are used to detect colon cancer?	Yes	149	38.7%
	No	236	61.3%

**Table 2:** Awareness regarding CRC CRC: colorectal cancer *Chi-square test

		Total n=385		
		Have you ever heard of CRC?		
		Yes, n (%)	No, n (%)	p-value*
Family or close friends had a CRC history	Yes, n (%)	63 (85.1%)	11 (14.9%)	.002
	No, n (%)	209 (67.2%)	102 (32.8%)	

Table 3 shows that participants with family or close friends with a history of CRC had heard more about CRC screening tests than other participants (p=.000). Among the 385 participants, only 38.7% had heard about the colorectal screening test.

**Table 3:** Knowledge and awareness of colorectal cancer screening CRC: colorectal cancer *Chi-square test

		Total n=385		
		Have you ever heard of any screening test used to detect CRC?		
		Yes, n (%)	No, n (%)	p-value*
Family or close friends had a CRC history	Yes, n (%)	43 (58.1%)	31 (41.9%)	.000
	No, n (%)	106 (38.7%)	205 (61.3% )	

The level of knowledge regarding colorectal cancer was unsatisfactory since 63.4% showed an insufficient level of knowledge (non-tabulated data). As shown in Table 4, the current study investigated the association between participants' demographic factors and their knowledge about CRC. Apparently, most demographic factors showed an association with the participants' knowledge regarding CRC. For example, age, educational status, and region of residence all showed a significant association with the knowledge score (p-value <.001, .002, <.001, respectively). Additionally, a health care career was significantly associated with the participants' knowledge about CRC (p=.002). However, the association between having a family member or a friend who was diagnosed with CRC and the knowledge score failed to achieve statistical significance (p=.064).

**Table 4:** Factors associated with CRC knowledge CRC: colorectal cancer *Chi-square test

Total n=385		Knowledge score				
		Insufficient		Sufficient		
		n	%	n	%	p-value*
Age (years)	18-24	64	26.2%	72	51.1%	˂.001
	25-40	110	45.1%	39	27.7%	
	41-50	50	20.5%	21	14.9%	
	>50	20	8.2%	9	6.4%	
Gender	Male	65	26.6%	26	18.4%	.068
	Female	179	73.4%	115	81.6%	
Education	University degree level	177	72.5%	122	86.5%	.002
	Below university degree level	67	27.5%	19	13.5%	
Career related to health care	Related to health care	39	16.0%	41	29.1%	.002
	Not related to health care	205	84.0%	100	70.9%	
Region	Taif	93	38.1%	89	63.1%	˂.001
	Makkah	57	23.4%	25	17.7%	
	Jeddah	40	16.4%	17	12.1%	
	Medina	45	18.4%	8	5.7%	
	Yanbu	9	3.7%	2	1.4%	
Family or close friends had a CRC history	Yes	40	16.4%	34	24.1%	.064
	No	204	83.6%	107	75.9%	
Have you ever heard of CRC?	Yes	145	59.4%	127	90.1%	˂.001
	No	99	40.6%	14	9.9%	
Have you ever heard of any screening tests that are used to detect colon cancer?	Yes	49	20.1%	100	70.9%	˂.001
	No	195	79.9%	41	29.1%	

**Figure 1:**
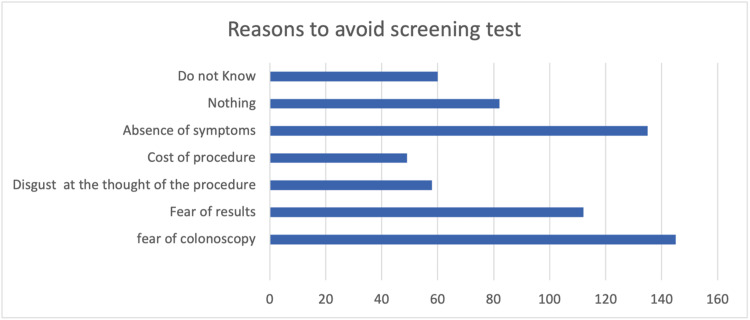
Reasons to avoid the screening test

Figure 1 shows the findings of reported reasons by the study participants that prevented them from having a screening test for CRC. The fear of colonoscopy and the absence of symptoms were the top reasons reported. On the other hand, the cost of the procedure was the least reported reason.

## Discussion

Colorectal cancer is the second most common cause of cancer mortality in the United States [9]. However, its mortality risk and cure rate are getting progressively better due to the early detection, and thus early cure is possible [10]. According to Siegel et al., the cumulative lifetime risk for developing colon cancer in men is 1 in 20, while it is 1 in 22 in women [11]. Screening for CRC is crucial for early detection and treatment as well as possible prevention by identifying and resecting precancerous polyps [12]. As per the US Preventive Services Task Force (USPSTF) guidelines, CRC screening is recommended for average-risk asymptomatic adults between the ages of 50 and 75 using either colonoscopy every 10 years, flexible sigmoidoscopy every five years, or computed tomography (CT) colonography every five years [13-14]. However, for patients at high risk, such as those with a family history of CRC in a first-degree relative, screening should be started 10 years before the age of diagnosis of the affected family member [14].

A total of 385 participants were involved in this study, most of whom were aged 25-40 years old, and around three-quarters of the participants were females. Furthermore, the majority of them had got university-level education and did not work in the health care field. In terms of the knowledge of CRC, around 70% of participants had heard about CRC, which is similar to the results of another study done by Saeed et al. about the knowledge and awareness of CRC in Kuwait in which they found out that approximately 75% of participants had heard about CRC at some point of their lives [15]. Moreover, a higher knowledge score was significantly associated with an age of 18-24 and a higher educational degree with a p-value of <0.001 and 0.002, respectively. This is consistent with what was concluded in a study by Lewandowski et al. about CRC knowledge and awareness [16]. However, according to another research, no significant association was found between the knowledge of CRC and age, occupation, or educational level [17].

As for CRC screening, around 38% of participants had heard about CRC screening and its tests, which is better compared to another similar study in which only 24% of participants knew about CRC screening tests. These people had been found to have better knowledge and understanding of CRC signs and symptoms [17]. Regarding the reasons for which participants may avoid screening, the most commonly reported reason among participants involved in this research was the fear of colonoscopy followed by an absence of symptoms that would raise suspicion or fear of CRC. This is in line with what was found in the literature, as the most commonly reported causes of avoiding CRC screening are being afraid and not having any symptoms at all [18]. On the other hand, another cross-sectional study that was carried out in China in 2019, reported having no symptoms or gastrointestinal discomfort as the most common reported cause for not undergoing colonoscopy or screening [19]. Other reported reasons for avoiding screening tests for CRC were pain, anxiety, and lack of time [20]. Therefore, the relationship between CRC awareness and screening is an essential aspect that must be emphasized. As per Brandt et al. in their cross-sectional study regarding this relationship, it was concluded that having more comprehensive knowledge and awareness regarding CRC as a disease was associated with a better attitude towards CRC, as more knowledgeable subjects were found to be more convinced to perform the requested screening tests compared to less knowledgeable people [21]. In fact, what further implies the lack of awareness and need for proper public health education regarding this matter is that according to the results of a cross-sectional study that was carried out in China involving 684 participants, only around 40% of the participants knew that CRC can be asymptomatic [22]. Thus, seeking medical assistance usually takes place when patients are symptomatic, which can imply an advanced stage of the disease [23].

Therefore, awareness of CRC and its screening tests should be better as it is associated with a better understanding of the disease and its potential signs and symptoms. Furthermore, the procedure should be explained clearly to all in order to ease their anxiety and fear regarding CRC screening tests.

Although the current study adds a valuable contribution to the local literature, further studies are suggested to overcome certain limitations this study encountered. A more representative sample can be obtained through a probability sampling technique and a wide-range sampling frame involving various regions in Saudi Arabia. Moreover, an interview data collection technique may assess the participants' knowledge better, and hence yield accurate findings.

## Conclusions

Colorectal cancer is ranked as one of the most prevalent cancer types worldwide and locally in Saudi Arabia. The consequent complications of such a serious condition are devastating, resulting in high morbidity and mortality. However, colorectal cancer is a preventable disease with acceptable screening methods. The current study revealed certain factors such as fear of colonoscopy and absence of symptoms as reasons to avoid CRC screening. Moreover, insufficient knowledge among the participants was associated with older age and below university degree level of education. Thus, awareness-raising campaigns should target these categories, especially the older age group, as they are at a higher risk of developing colorectal cancer and are fit for the screening tests.
